# Cancer Fatalism, Literacy, and Cancer Information Seeking in the American Public

**DOI:** 10.1177/1090198115604616

**Published:** 2015-09-16

**Authors:** Lindsay C. Kobayashi, Samuel G. Smith

**Affiliations:** 1 University College London, London, UK; 2 Queen Mary University of London, London, UK

**Keywords:** cancer, communication, fatalism, Health Information and National Trends Survey (HINTS), health literacy, information seeking

## Abstract

Information seeking is an important behavior for cancer prevention and control, but inequalities in the communication of information about the disease persist. Conceptual models have suggested that low health literacy is a barrier to information seeking, and that fatalistic beliefs about cancer may be a mediator of this relationship. Cancer fatalism can be described as deterministic thoughts about the external causes of the disease, the inability to prevent it, and the inevitability of death at diagnosis. This study aimed to examine the associations between these constructs and sociodemographic factors, and test a mediation model using the American population-representative Health Information and National Trends Survey (HINTS 4), Cycle 3 (*n* = 2,657). Approximately one third (34%) of the population failed to answer 2/4 health literacy items correctly (limited health literacy). Many participants agreed with the fatalistic beliefs that it seems like everything causes cancer (66%), that one cannot do much to lower his or her chances of getting cancer (29%), and that thinking about cancer makes one automatically think about death (58%). More than half of the population had “ever” sought information about cancer (53%). In analyses adjusted for sociodemographic characteristics and family cancer history, people with limited health literacy were less likely to have ever sought cancer information (odds ratio [*OR*] = 0.63; 0.42-0.95) and more frequently endorsed the belief that “there’s not much you can do . . .” (*OR* = 1.61; 1.05-2.47). This fatalistic belief partially explained the relationship between health literacy and information seeking in the mediation model (14% mediation). Interventions are needed to address low health literacy and cancer fatalism to increase public interest in cancer-related information.

In 2014, there were an estimated 1.6 million new cancer cases and 580,000 cancer deaths in the United States (Siegel, Ma, Zou, & Jemal, 2014). At least one third of these cancers are preventable through behaviors such as not smoking, engaging in physical activity, maintaining a healthy diet, and adhering to cancer screening guidelines (Parkin, 2011; Vineis & Wild, 2014). There is marked room for improvement in uptake of health behaviors and cancer screening in the American population. For example, 18% of the adult American population smoke (Centers for Disease Control and Prevention, 2015), more than half do not meet national physical activity guidelines (Centers for Disease Control and Prevention, 2014), and uptake of some cancer screening modalities is low (Centers for Disease Control and Prevention, 2013). Information seeking may be one route through which individuals improve their ability to make informed decisions, and engage more readily in behavior change throughout the continuum of cancer prevention, early diagnosis, treatment, and survival (Anker, Reinhart, & Feeley, 2011; Ramanadhan & Viswanath, 2006; Ramírez et al., 2013; Rutten, Arora, Bakos, Aziz, & Rowland, 2005; Viswanath et al., 2012).

Cancer information seeking in the population is increasing, but the rises observed over the past decade have been greater among those with higher levels of education and income (Finney Rutten et al., 2015). This trend suggests that communication inequalities are widening, and will likely continue to do so (Viswanath, 2005). Health literacy may play a role in explaining socioeconomic inequalities in cancer-related information seeking (Paasche-Orlow & Wolf, 2007; von Wagner, Steptoe, Wolf, & Wardle, 2009). Health literacy represents functional literacy skills in health contexts, and is defined as “the capacity to obtain, process, and understand basic health information and services needed to make appropriate health decisions” (Institute of Medicine, 2004). One in three American adults possesses low health literacy, the burden of which is disproportionately held by older adults, racial/ethnic minorities, the less educated, and those with low household incomes (Kutner, Greenberg, Jin, Paulsen, & White, 2007).

Conceptual health literacy frameworks have built on existing psychological theories to map pathways linking health literacy with sociocognitive factors (e.g., knowledge and beliefs), and in turn with behavioral outcomes such as screening, preventive behaviors, and information seeking (Paasche-Orlow & Wolf, 2007; von Wagner et al., 2009). These proposed pathways are gaining empirical support, with evidence that adults with low health literacy are less informed about cancer (Boxell et al., 2012; Morris et al., 2013; Smith, Forster, & Kobayashi, 2015), have more negative beliefs about prevention, early diagnosis, and treatment (Dolan et al., 2004; Smith, Kobayashi, et al., 2014), and are less likely to adhere to prevention recommendations (Kobayashi, Wardle, & von Wagner, 2014; von Wagner, Knight, Steptoe, & Wardle, 2007). A simplified version of these conceptual models forms the basis for this study and is presented in Figure 1.

**Figure 1.**
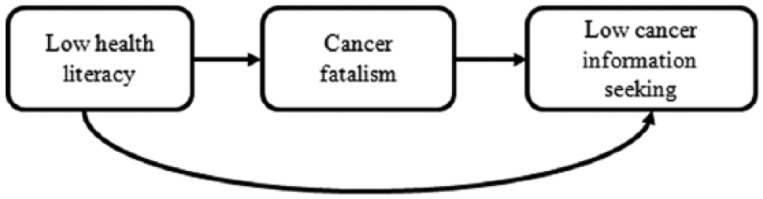
Conceptual model linking health literacy, cancer fatalism, and information seeking.

Fatalism is a particularly salient belief to consider in the context of these models because of its association with cancer-related health behaviors (Niederdeppe & Levy, 2007; Powe & Finnie, 2003), delays in symptomatic presentation (Beeken, Simon, von Wagner, Whitaker, & Wardle, 2011; Lyratzopoulos, Liu, Abel, Wardle, & Keating, 2015), and avoidance of cancer information (Miles, Voorwinden, Chapman, & Wardle, 2008). Cancer fatalism can include deterministic thoughts about the external causes of cancer, the inability to prevent it, and the inevitability of death at diagnosis (Niederdeppe & Levy, 2007). Cancer fatalism may be an outcome of living and coping with experiences that invoke hopelessness and despair (Powe & Finnie, 2003). People with low health literacy may be more likely to observe poorer cancer outcomes firsthand within their social environments (Davis, Williams, Marin, Parker, & Glass, 2002), and it therefore seems reasonable to hypothesize that they will be more fatalistic and less likely to pursue additional information about the disease.

Using data from the 2013 U.S. Health Information and National Trends Survey (HINTS) 4 Cycle 3, we aimed to (a) investigate the associations between sociodemographic factors, health literacy, cancer fatalism, and cancer information seeking in the adult American population and (b) examine whether cancer fatalism mediates the relationship between health literacy and cancer information seeking. Based on existing evidence and theoretical models, we hypothesized that people with limited health literacy and who hold fatalistic beliefs about cancer would be less likely to seek cancer information, and that the relationship between health literacy and cancer information seeking would be at least partly mediated by cancer fatalism.

## Method

### Data Source

Data were from the third cycle of the fourth HINTS. The HINTS is a national probability-based survey of U.S. adults established in 2003 and conducted by the National Cancer Institute to study public attitudes and behaviors associated with cancer. Data for the third cycle were collected from September 2013 through December 2013. A description of the sampling and recruitment procedures is available elsewhere (Westat, 2014).

A total of 3,185/12,010 people returned completed surveys through the mail (27% response rate). We excluded people who reported uncertainty about their cancer history or a previous cancer diagnosis (*n* = 508) and those who received a Spanish-language questionnaire that did not include the literacy assessment (*n* = 20), leaving an analyzable sample of 2,657 English-speaking U.S. adults who reported no history of cancer. Informed consent was obtained from all individual participants included in the study.

### Measures

#### Health Literacy

The Newest Vital Sign (NVS; Weiss et al., 2005) is a commonly used six-item health literacy assessment (Berkman, Sheridan, Donahue, Halpern, & Crotty, 2011). A four-item short-form version of the NVS was used in this study. Respondents are asked to read a nutritional label of an ice cream container and answer four health-related reading comprehension and numeracy questions. One point is allocated for each correct answer and different thresholds for “adequate” health literacy were tested.

#### Fatalism

Respondents answered the following statements on a 4-point Likert-type scale ranging from *strongly agree* to *strongly disagree*: “It seems like everything causes cancer” (Belief 1), “There’s not much you can do to lower your chances of getting cancer” (Belief 2), and, “When I think about cancer, I automatically think about death” (Belief 3). The fatalism items are unique to the HINTS surveys; they have been used in multiple years and show predictive capability for cancer preventive health behaviors (Niederdeppe & Levy, 2007). Agreement was defined as responding *strongly agree* or *somewhat agree* (Niederdeppe & Levy, 2007). The three beliefs were significantly, but weakly correlated: Spearman’s *r*_1, 2_ = 0.31, *r*_1, 3_ = 0.27, and *r*_2, 3_ = 0.22 (all *p* < .0001). Cronbach’s alpha for the three items was low (α = .52), indicating that they should not be used as a scale.

#### Cancer Information Seeking

A single-item measure of cancer information seeking was asked, “Have you ever looked for information about cancer from any source?” (yes; no).

#### Participant Characteristics

Measures of age, gender, educational attainment (<high school; high school; some college; bachelor’s degree; postbaccalaureate), household income (<$20,000; $20,000-34,999; $35,000-49,999; $50,000-74,999; $75,000+), race/ethnicity (Non-Hispanic White; Hispanic; Non-Hispanic Black; Other Non-Hispanic), marital status (single; married, or living as married), and family cancer history (yes; no; unsure) were recorded.

### Statistical Analysis

To establish an appropriate threshold for “limited health literacy” using the short-form NVS, we tested thresholds of 1, 2, 3, and 4 incorrect items out of 4. The weighted proportion of respondents classified as having limited health literacy according to each threshold was compared with those from a nationally representative prevalence estimate and a systematic review of American health literacy studies (Kutner et al., 2007; Paasche-Orlow, Parker, Gazmararian, Nielsen-Bohlman, & Rudd, 2005). The threshold giving the closest figure to these estimates was selected for analysis. Limited health literacy (dichotomous) was used for all analyses involving proportions and sociodemographic associations, as it is intended to be a meaningful threshold for public health and education purposes. In contrast, the mediation analysis uses the health literacy score out of 4 as the continuous independent variable, to capture variation in health literacy that may influence cancer fatalism and information seeking. The mediation analysis was repeated using the dichotomous limited health literacy variable and no changes in the results were noted (data not shown).

**Figure 2.**
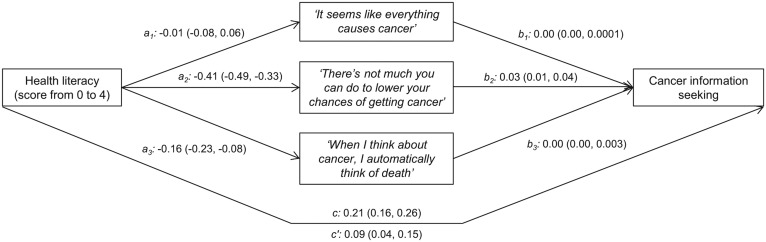
Model investigating the direct and indirect effects between health literacy, cancer fatalism, and information seeking.

Data were weighted to ensure representativeness of the adult American population (Westat, 2014). Weighted proportions of limited health literacy, agreement with the three fatalistic beliefs, and cancer information seeking were calculated overall and by participant characteristics. Weighted logistic regression models adjusted for all covariates estimated associations between sociodemographic characteristics and each of limited health literacy, cancer fatalism, and cancer information seeking. As outlined in Figure 2, mediation analysis was performed using Kenny’s binary outcome method to estimate standardized coefficients for (a) the direct effect of health literacy on each fatalistic belief (paths *a*_1_*-a*_3_), (b) the indirect effects of health literacy on cancer information seeking through each fatalistic belief (paths *b*_1_*-b*_3_), (c) the direct, unadjusted effect of health literacy on cancer information seeking (*c*), (d) the direct effect of health literacy on cancer information seeking, independent of the fatalistic beliefs and covariates (*c′*), and (e) the proportion of the total effect of health literacy score on information seeking that was mediated by the fatalistic beliefs (Herr, 2014; Kenny, 2014). The mediation model could not be weighted, but was adjusted for sociodemographic and other covariates. Bias-corrected 95% confidence intervals (CIs) were estimated for all coefficients via bootstrapping with 500 replications. All analyses were performed using Stata 13.1 (StataCorp, College Station, TX).

## Results

### Health Literacy

When using thresholds of one, two, three, and four incorrect answers, respectively, to define “limited” health literacy, the proportions with limited health literacy were 58% (95% CI: 55% to 61%), 34% (31% to 36%), 19% (17% to 21%), and 8% (7% to 9%). The threshold of two incorrect answers (34%) gave a proportion closest to estimates in a nationally representative survey (36%; Kutner et al., 2007) and a systematic review (46%; Paasche-Orlow et al., 2005), and was therefore selected for subsequent analyses. Having limited health literacy (according to the cutoff of two incorrect answers) was associated with older age, low education, low income, and non-Hispanic Black and Hispanic race/ethnicity (Table 1).

**Table 1.** Weighted Participant Characteristics, Overall and by Limited Health Literacy, HINTS 4 Cycle 3, 2013, *n* = 2,657.

	Overall	Limited health literacy	
Characteristic (unweighted *n*)	% (95% CI)	% (95% CI)	*OR* (95% CI) unweighted *n* = 2,021
Age in years (*n* = 2,592)			
18-34	29 (28, 31)	24 (19, 31)	1.0 (reference)
35-49	32 (30, 34)	35 (29, 41)	1.89 (0.97, 3.68)
50-64	25 (24, 25)	29 (26, 33)	1.55 (0.89, 2.69)
65-74	8 (7, 8)	44 (38, 51)	3.27 (1.68, 6.34)^†^
≥75	6 (6, 7)	59 (52, 67)	5.08 (2.30, 11.20)^†^
Sex (*n* = 2,611)			
Male	49 (48, 50)	33 (28, 37)	1.0 (reference)
Female	51 (50, 52)	33 (30, 36)	0.84 (0.62, 1.14)
Educational attainment (*n* = 2,612)			
Postbaccalaureate	13 (11, 14)	17 (12, 22)	1.0 (reference)
Bachelor’s degree	21 (19, 22)	24 (19, 30)	1.47 (0.81, 2.67)
Some college	33 (31, 35)	24 (20, 28)	1.63 (0.78, 3.39)
High school	24 (22, 26)	44 (38, 51)	3.17 (1.64, 6.14)^†^
Less than high school	9 (8, 11)	78 (70, 84)	7.41 (2.98, 18.40)^†^
Household income in $ (*n* = 2,318)			
≥75,000	33 (30, 36)	17 (13, 22)	1.0 (reference)
50,000-74,999	18 (15, 20)	30 (23, 39)	1.78 (0.96, 3.32)
35,000-49,999	15 (12, 18)	26 (19, 33)	0.98 (0.59, 1.62)
20,000-34,999	14 (12, 17)	40 (33, 47)	1.36 (0.85, 2.16)
<$20,000	20 (18, 23)	54 (46, 62)	2.76 (1.60, 4.77)^†^
Race/ethnicity (*n* = 2,323)			
Non-Hispanic White	66 (65, 67)	20 (17, 23)	1.0 (reference)
Hispanic	16 (15, 16)	51 (45, 57)	3.63 (2.31, 5.71)^†^
Non-Hispanic Black	11 (10, 12)	60 (49, 70)	5.60 (3.16, 9.91)^†^
Other non-Hispanic	8 (7, 8)	32 (23, 42)	2.16 (1.08, 4.32)*
Marital status (*n* = 2,599)			
Married/cohabiting	58 (57, 60)	29 (27, 32)	1.0 (reference)
Single	42 (40, 43)	38 (33, 42)	1.14 (0.77, 1.70)
Family cancer history (*n* = 2,563)			
Yes	65 (62, 68)	28 (25, 32)	1.0 (reference)
No	26 (23, 28)	40 (36, 44)	1.30 (0.86, 1.97)
Not sure	9 (7, 11)	42 (31, 55)	1.61 (0.66, 3.90)

*Note*. CI = confidence interval; *OR* = odds ratio; HINTS = Health Information and National Trends Survey. All variables in the left column are adjusted for in the model. Limited health literacy was defined as scoring 2 or fewer out of 4 items correct on the assessment.

* *p* < .05. ^†^*p* < .001.

### Cancer Fatalism

Table 2 shows the overall distribution of agreement with the three fatalistic cancer beliefs, according to the 4-point Likert-type scale. When the scale was dichotomized to show agreement versus disagreement with the beliefs, most people agreed (66%; 63%-70%) with the belief “It seems like everything causes cancer” (Belief 1), whereas most disagreed (71%; 68%-74%) that “There’s not much you can do to lower your chances of getting cancer” (Belief 2). Responses to the item, “When I think about cancer, I automatically think about death” (Belief 3) were more evenly balanced with 42% (38% and 45%) disagreeing and 58% (55% to 62%) agreeing.

**Table 2.** Weighted Percent distribution (95% CI) of Responses to the Cancer Fatalism Items, HINTS 3 Cycle 4, 2013, *n* = 2,657.

Fatalistic belief	Strongly agree	Somewhat agree	Somewhat disagree	Strongly disagree
“. . . everything causes cancer”	20 (17, 23)	47 (43, 50)	20 (18, 23)	13 (12, 15)
“. . . not much you can do”	7 (5, 9)	22 (20, 25)	41 (38, 45)	30 (27, 33)
“. . . think of death”	19 (17, 23)	39 (36, 43)	27 (24, 31)	14 (12, 17)

*Note*. CI = confidence interval; HINTS = Health Information and National Trends Survey.

The relationships between participant characteristics, including health literacy, and each of fatalistic beliefs and cancer information seeking are shown in Table 3 (weighted proportions) and Table 4 (adjusted, weighted odds ratios). Agreement with Belief 1 was associated with younger age, female sex, and low education, but not with health literacy (Tables 3 and 4). Agreement with Belief 2 was associated with low education, low income, non-Hispanic Black race, single marital status, and limited health literacy (Tables 3 and 4). Agreement with Belief 3 was only associated with younger age (Tables 3 and 4).

**Table 3.** Weighted Proportions of Cancer Fatalism and Information Seeking by Sociodemographic and Other Factors, HINTS 4 Cycle 3, 2013, *n* = 2,657.

Characteristic	Agrees “. . . everything causes cancer”	Agrees “. . . not much you can do”	Agrees “. . . think of death”	Cancer information seeking
	% (95% CI)	% (95% CI)	% (95% CI)	% (95% CI)
Overall	66 (63, 70)	29 (26, 32)	58 (55, 62)	53 (50, 56)
Health literacy				
Adequate	68 (64, 72)	23 (19, 26)	55 (51, 60)	59 (55, 63)
Limited	64 (58, 69)	42 (37, 46)	65 (60, 70)	40 (36, 45)
*p* value	.23	<.0001	.002	<.0001
Age (years)				
18-34	75 (67, 81)	29 (23, 35)	58 (51, 65)	52 (45, 58)
35-49	63 (56, 70)	29 (23, 36)	62 (56, 69)	54 (48, 59)
50-64	67 (62, 72)	25 (21, 29)	56 (51, 61)	58 (52, 64)
65-74	59 (51, 67)	34 (28, 41)	61 (54, 67)	55 (49, 61)
≥75	55 (43, 65)	33 (25, 42)	49 (38, 60)	38 (31, 46)
*p* value	.001	.37	.18	.04
Sex				
Male	62 (57, 67)	31 (26, 36)	59 (53, 65)	49 (45, 54)
Female	70 (66, 74)	27 (24, 30)	58 (54, 62)	57 (43, 60)
*p* value	.005	.24	.84	.01
Educational attainment				
Postbaccalaureate	53 (47, 59)	16 (12, 23)	50 (42, 58)	71 (65, 76)
Bachelor’s degree	61 (56, 67)	22 (17, 29)	57 (51, 63)	61 (55, 66)
Some college	74 (68, 79)	24 (19, 30)	60 (53, 66)	56 (51, 62)
High school	72 (66, 77)	42 (36, 47)	61 (53, 69)	42 (36, 48)
Less than high school	56 (45, 66)	41 (31, 52)	64 (54, 73)	30 (22, 40)
*p* value	<.0001	<.0001	.18	<.0001
Household income ($)				
≥75,000	67 (63, 72)	20 (15, 27)	55 (48, 61)	63 (57, 69)
50,000-74,999	62 (53, 70)	30 (24, 38)	59 (50, 68)	58 (50, 66)
35,000-49,999	67 (57, 75)	23 (17, 30)	51 (41, 61)	58 (48, 68)
20,000-34,999	73 (64, 80)	37 (29, 46)	60 (49, 71)	39 (32, 47)
<20,000	64 (56, 72)	39 (32, 46)	67 (60, 73)	40 (34, 47)
*p* value	.40	.0001	.12	<.0001
Race/ethnicity				
Non-Hispanic White	69 (65, 74)	24 (21, 28)	57 (52, 61)	56 (52, 60)
Hispanic	63 (55, 70)	30 (24, 36)	60 (51, 67)	46 (39, 52)
Non-Hispanic Black	58 (47, 68)	39 (30, 47)	67 (57, 75)	52 (43, 61)
Other non-Hispanic	67 (57, 75)	37 (24, 51)	55 (43, 67)	56 (44, 67)
*p* value	.08	.005	.26	.12
Marital status				
Single	66 (60, 71)	28 (24, 34)	59 (53, 65)	47 (41, 52)
Married/cohabiting	67 (63, 70)	29 (25, 33)	58 (54, 62)	58 (54, 61)
*p* value	.85	.93	.76	.0015
Family cancer history				
No	58 (53, 64)	31 (26, 37)	59 (52, 65)	38 (31, 44)
Yes	69 (65, 73)	26 (23, 29)	57 (53, 61)	62 (59, 66)
Not sure	65 (52, 76)	41 (29, 54)	67 (55, 77)	34 (24, 45)
*p* value	.02	.02	.20	<.0001

*Note*. HINTS = Health Information and National Trends Survey. “Agree” refers to combined responses of “strongly agree” and “somewhat agree.” Limited health literacy was defined as scoring 2 or fewer out of 4 items correct on the assessment.

**Table 4.** Weighted Multivariable Associations Between Health Literacy, Sociodemographic Characteristics, and Cancer Fatalism and Information Seeking, HINTS 4 Cycle 3, 2013, *n* = 2,657.

Characteristic	Agrees “. . . everything causes cancer”	Agrees “. . . not much you can do”	Agrees “. . . automatically think of death”	Cancer information seeking
	*OR* (95% CI)	*OR* (95% CI)	*OR* (95% CI)	*OR* (95% CI)
Health literacy				
Adequate	1.0 (reference)	1.0 (reference)	1.0 (reference)	1.0 (reference)
Limited	1.01 (0.67, 1.53)	1.61 (1.05, 2.47)*	1.39 (0.99, 1.95)	0.63 (0.42, 0.95)*
Age (years)				
18-34	1.0 (reference)	1.0 (reference)	1.0 (reference)	1.0 (reference)
35-49	0.61 (0.34, 1.10)	0.84 (0.48, 1.47)	1.00 (0.65, 1.55)	1.27 (0.84, 1.93)
50-64	0.61 (0.36, 1.04)	0.66 (0.41, 1.08)	0.76 (0.53, 1.11)	1.38 (0.90, 2.12)
65-74	0.45 (0.24, 0.86)*	0.86 (0.51, 1.43)	0.97 (0.56, 1.70)	1.50 (0.88, 2.15)
≥75	0.36 (0.16, 0.80)*	0.78 (0.39, 1.57)	0.49 (0.24, 0.99)*	1.09 (0.60, 1.99)
Sex				
Male	1.0 (reference)	1.0 (reference)	1.0 (reference)	1.0 (reference)
Female	1.43 (1.07, 1.91)*	0.85 (0.56, 1.28)	0.96 (0.69, 1.35)	1.31 (0.99, 1.73)
Educational attainment				
Postbaccalaureate	1.0 (reference)	1.0 (reference)	1.0 (reference)	1.0 (reference)
Bachelor’s degree	1.46 (0.99, 2.16)	1.57 (0.80, 3.06)	1.17 (0.75, 1.82)	0.71 (0.48, 1.07)
Some college	2.42 (1.65, 3.56)^†^	2.01 (1.16, 3.48)*	1.26 (0.85, 1.88)	0.67 (0.42, 1.05)
High school	2.73 (1.69, 4.42)^†^	4.27 (2.02, 9.04)^†^	1.37 (0.80, 2.33)	0.39 (0.24, 0.63)^†^
Less than high school	1.45 (0.74, 2.83)	2.35 (0.94, 5.89)	1.39 (0.62, 3.13)	0.33 (0.15, 0.74)*
Household income ($)				
≥75,000	1.0 (reference)	1.0 (reference)	1.0 (reference)	1.0 (reference)
50,000-74,999	0.60 (0.39, 0.93)*	1.26 (0.70, 2.26)	1.09 (0.65, 1.85)	1.01 (0.66, 1.54)
35,000-49,999	0.70 (0.41, 1.20)	0.84 (0.46, 1.53)	0.79 (0.43, 1.42)	0.92 (0.51, 1.66)
20,000-34,999	1.17 (0.67, 2.03)	1.86 (1.03, 3.36)*	1.15 (0.62, 2.11)	0.51 (0.28, 0.93)*
<20,000	0.71 (0.39, 1.27)	1.52 (0.79, 2.94)	1.37 (0.77, 2.43)	0.61 (0.32, 1.16)
Race/ethnicity				
Non-Hispanic White	1.0 (reference)	1.0 (reference)	1.0 (reference)	1.0 (reference)
Hispanic	0.83 (0.57, 1.19)	0.80 (0.54, 1.19)	0.94 (0.61, 1.45)	1.49 (0.95, 2.32)
Non-Hispanic Black	0.69 (0.38, 1.27)	1.69 (1.05, 2.72)*	1.17 (0.70, 1.97)	1.60 (0.94, 2.73)
Other non-Hispanic	1.14 (0.66, 1.99)	1.93 (0.90, 4.13)	0.80 (0.44, 1.45)	1.22 (0.65, 2.32)
Marital status				
Married/cohabiting	1.0 (reference)	1.0 (reference)	1.0 (reference)	1.0 (reference)
Single	0.85 (0.55, 1.32)	0.65 (0.42, 0.99)*	0.96 (0.67, 1.39)	0.74 (0.52, 1.05)
Family cancer history				
Yes	1.0 (reference)	1.0 (reference)	1.0 (reference)	1.0 (reference)
No	0.76 (0.55, 1.04)	1.07 (0.75, 1.54)	1.14 (0.78, 1.67)	0.33 (0.22, 0.50)^†^
Not sure	0.92 (0.48, 1.79)	1.89 (0.91, 3.95)	1.37 (0.68, 2.75)	0.33 (0.18, 0.59)^†^
Analytic sample (unweighted)	1,995	1,989	1,988	2,005

*Note*. CI = confidence interval; *OR* = odds ratio; HINTS = Health Information and National Trends Survey. All variables in the left column are adjusted for in all models.

* *p* < .05. ^†^*p* < .001.

### Cancer Information Seeking

More than half of the population had ever sought information about cancer (53%; 50%-56%). Those with lower education, lower income, and no or uncertain family histories of cancer had lower odds of seeking cancer information (Tables 3 and 4). Forty percent (36%-45%) of those with limited health literacy reported seeking cancer information, compared with 59% (55%-63%) of those with adequate health literacy (*OR* = 0.63; 0.42-0.95; Tables 3 and 4). Table 5 shows the associations between the three fatalistic beliefs and cancer information seeking. The belief that everything causes cancer (Belief 1) and automatic thoughts of death (Belief 3) were not associated with cancer information seeking in multivariable-adjusted logistic regression models, but those who believed that there’s not much you can do to lower your risk of cancer (Belief 2) had lower odds of seeking cancer information (*OR* = 0.63; 0.43-0.93).

**Table 5.** Weighted Multivariable-Adjusted Logistic Regression Models Predicting Cancer Information Seeking, HINTS 4 Cycle 3, 2013.

Fatalistic belief	Cancer information seeking; OR (95% CI)	Unweighted *n*
Agrees “. . . everything causes cancer”	1.01 (0.71, 1.43)	1,981
Agrees “. . . not much you can do”	0.63 (0.43, 0.93)	1,974
Agrees “. . . automatically think of death”	0.80 (0.58, 1.12)	1,973

*Note*. HINTS = Health Information and National Trends Survey; OR, odds ratio; CI, confidence interval. “Agree” refers to combined responses of “strongly agree” and “somewhat agree.” All models adjusted for age, sex, education, household income, race/ethnicity, marital status, and family cancer history.

### Mediation Analysis

Figure 2 shows the results of the mediation analysis. The total direct effect of health literacy score on cancer information seeking was 0.21 (0.16-0.26). Health literacy score was not associated with Belief 1, but was inversely associated with agreement with Belief 2 (−0.41; −0.49 to −0.33) and Belief 3 (−0.16; −0.23 to −0.08). Beliefs 1 and 3 were not independently associated with cancer information seeking, explaining their lack of mediation of health literacy effects. However, Belief 2, “There’s not much you can do to lower your chances of getting cancer,” mediated 14% of the effect of health literacy on cancer information seeking (indirect effect = 0.03; 0.02-0.05). The remaining direct effect of health literacy on cancer information seeking, after accounting for cancer fatalism and sociodemographic factors, was 0.09 (0.04-0.15), representing 43% of its effect. Independently, Belief 2 had the strongest effect on cancer information seeking than any other variable examined in this analysis (−0.47; −0.69 to −0.25). The results for this analysis were similar when limited health literacy rather than health literacy score was used as the independent variable (not shown).

## Discussion

Despite consistent evidence-based cancer prevention recommendations and increasing rates of cancer survival in the population, these nationally representative data indicate that two thirds of the U.S. population felt like everything causes cancer, one third did not believe cancer is preventable, and more than half automatically associated cancer with death. Together with low health literacy, believing that cancer is not preventable was associated with a lower likelihood of seeking cancer information. Somewhat in support of our conceptual framework, this fatalistic belief accounted for a small part of the association between health literacy and cancer information seeking. Addressing health literacy and fatalism about cancer prevention should be a priority for future cancer communication strategies, particularly for those targeting medically underserved population groups.

Although sociodemographic associations varied across the individual fatalistic beliefs, they were particularly common among younger adults, women, those with low education, low income, non-Hispanic Black adults, single adults, and those with low health literacy. Similar socioeconomic inequalities were noted with regard to cancer information seeking, consistent with previous research (Viswanath, 2005; Viswanath et al., 2012). Although reductive perspectives on the literacy skills and beliefs of people from socially deprived backgrounds are cautioned against, clinicians should be aware of these broad inequalities and be prepared to work with their patients to promote learning about cancer control. Physician guidelines for improving communication with patients who have low literacy are available, and should be incorporated into medical education (Kripalani & Weiss, 2006).

However, strategies to improve beliefs and knowledge about cancer in the population may be best placed outside the clinical environment. Events in opportunistic settings such as roadshows may help increase incidental exposure to cancer information among those who are less likely to actively seek it (Alcaraz, Weaver, Andresen, Christopher, & Kreuter, 2011; Smith, Rendell, George, & Power, 2014). Future research should consider whether such events create “teachable moments” in which negative beliefs can be challenged and redefined to represent more accurate perceptions of cancer prevention, control, and treatment. Advertising campaigns, which encourage awareness about and action on symptoms of cancer through clear, simple messages, have been shown to be effective in the United Kingdom (Ironmonger et al., 2015; Power & Wardle, 2015).

Despite the association between fatalism and important cancer-related outcomes (Beeken et al., 2011; Lyratzopoulos et al., 2015), the construct of fatalism remains poorly defined, making the development of behavioral interventions difficult (Powe & Finnie, 2003). However, some strategies have been successful. For example, a single-arm pilot trial of a culturally targeted health education leaflet conducted among Black men in New York City was effective in reducing fatalism according to the 15-item Powe Fatalism Inventory, and these improvements predicted participation in cancer screening at follow-up (Philip, DuHamel, & Jandorf, 2010). Patient narratives, such as positive stories from cancer survivors, may also be an effective strategy. Experiences from breast cancer survivors have been shown to improve engagement with the topic of mammography, and to reduce counterarguing and fatalistic beliefs (McQueen, Kreuter, Kalesan, & Alcaraz, 2011). Narrative interventions show promise, and future research should continue to evaluate their effects on knowledge, beliefs, and information seeking.

Consistent with previous HINTS research, people with less education were more likely to hold the fatalistic beliefs that everything causes cancer and that it cannot be prevented (Niederdeppe & Levy, 2007). Independently of education, people in our study with limited health literacy were also less likely to believe cancer is preventable. This finding highlights that although health literacy and education are overlapping constructs, literacy accounts for additional explanatory variance in fatalism over and above education. Improved access to high-quality health education in schools and adult learning programs, in addition to improved communication from health providers should be advocated for in order to equalize opportunities to gain health literacy skills and health knowledge. Ultimately, this policy-level change would ideally also have positive effects on racial/ethnic and socioeconomic disparities in cancer outcomes.

These findings add some empirical support for the relationships between health literacy, fatalism, and cancer information seeking as outlined in conceptual health literacy frameworks (von Wagner et al., 2011; Paasche-Orlow & Wolf, 2007). However, the mediating effect of fatalism was only partial, emphasizing that dispositional characteristics such as blunting and coping style, as well as other attitudes and beliefs about cancer may be important (Anker et al., 2011; Case, Andrews, Johnson, & Allard, 2005). People who experience stronger emotional reactions to the threat of cancer may be less likely to search for information that conflicts with their existing beliefs in an attempt to avoid feelings of discomfort that arise during cognitive dissonance (Festinger, 1957). For example, cancer fear has been shown to predict information seeking independently of cancer fatalism (Miles et al., 2008). Identifying other emotional reactions to cancer that are prevalent among lower health literacy groups, and testing them within the conceptual model proposed here, may increase its explanatory power. While the opportunity to investigate all hypothesized factors in a single nationally representative data set is rare, investigators should consider ways to test more elaborative frameworks in future studies.

This study has limitations. Although our mediation analysis was hypothesis-driven, HINTS uses a cross-sectional design, which prevents causal inferences. Although we adjusted for important potentially confounding variables known to be associated with health literacy, cancer fatalism, and information seeking (Kelly et al., 2010; Niederdeppe & Levy, 2007; Paasche-Orlow et al., 2005), there may be unmeasured confounders that may affect fatalism, such as religiosity. Only single-item measures of cancer information seeking and of the three fatalism questions were available in this cycle of HINTS. Future rounds should attempt to include abbreviated, but validated scales of these constructs. The inclusion of the NVS measure within HINTS provided a rare opportunity to investigate health literacy in a nationally representative sample. However, the brief four-item version of the measure that was included means that variability in health literacy skills captured by this measure will be reduced compared with the original six-item measure. The HINTS was not designed to validate this new brief version, so we used previous nationally representative prevalence estimates to inform the selection of a cutoff point to define “limited health literacy.” The true prevalence of limited health literacy in the American population may differ if there have been changes in the population prevalence since the previous estimates were generated. Further validation of the brief NVS measure comparing in-person versus questionnaire administration and in comparison with other validated measures is required. Although the response rate to this cycle of HINTS was similar to previous years (27%), further efforts are needed to capture nonresponders in future cycles.

Cancer fatalism is prevalent in the U.S. population, and appears to be more common among adults with low health literacy. People with low health literacy skills were less likely to seek cancer information, and part of this association appears to be explained by the fatalistic belief that cancer cannot be prevented. Seeking information about cancer is an important behavior that enables further action on prevention and control; the ability to seek and access information should not be hindered unnecessarily by skills or beliefs. Fatalism and health literacy may represent useful targets for cancer control strategies aiming to increase the personal capacity of all individuals to manage their risk of cancer, and to reduce socioeconomic and racial disparities across the continuum of cancer control.
